# Supplementary material for: Asynchronous magnetic resonance elastography: Shear wave speed reconstruction using noise correlation of incoherent waves
Source: Magn Reson Med. 2022 Oct 27;89(3):990–1001. doi: 10.1002/mrm.29502 (PMC9792433; doi:10.1002/mrm.29502)
Supplement: Supplementary file 1 — DATA S1 MATLAB code used to generate the numerical simulations of Figure 1 [file MRM-89-990-s002.zip › k-Wave/helpfiles/flyThrough.html]

flyThrough :: Functions (k-Wave)


# flyThrough

Display a three-dimensional matrix slice by slice.

## Syntax

```
flyThrough(mat)
flyThrough(mat, dim)
flyThrough(mat, dim, fps)
flyThrough(mat, dim, fps, loop)
```

## Description

`flyThrough` produces an animated visualisation of the data within a three-dimensional matrix `mat` by displaying the matrix slice by slice using `imagesc` and the colormap returned by `getColorMap`. A constant scaling parameter is automatically computed using the maximum value within `mat`.

## Inputs

|  |  |
| --- | --- |
| `mat` | the three-dimensional matrix to visualise |

## Optional Inputs

|  |  |
| --- | --- |
| `dim` | matrix dimension through which the slices are taken (default = `1`) |
| `fps` | maximum number of frames per second to display (default = `5`) |
| `loop` | number of times to loop the animation (default = `1`) |

**Note**  The maximum achievable `fps` is determined by the speed at which the image slices can be extracted and displayed.

## Examples

- Simulations In Three-Dimensions
- 3D FFT Reconstruction For A Planar Sensor

## See Also

`getColorMap`, `imagesc`
